# Use of social media among Italian physiotherapists: a new opportunity for the profession or an unfavorable trend toward guruism?

**DOI:** 10.1186/s40945-016-0025-1

**Published:** 2016-09-20

**Authors:** Stefano Vercelli

**Affiliations:** Laboratory of Ergonomics and Musculoskeletal Disorders Assessment, Division of Physical Medicine and Rehabilitation, Salvatore Maugeri Foundation, Scientific Institute of Veruno, IRCCS, Via per Revislate 13, I-28010 Veruno (NO), Italy

**Keywords:** Web 2.0, Social Networking, Facebook, Health information systems

## Abstract

The advent of social media such as Facebook has introduced new opportunities for knowledge sharing and professional networking. Currently, little is known on how physiotherapists participate in virtual communities, and there are opposing views regarding the benefits and pitfalls of professional use of social media. In this letter, theoretical frameworks are proposed by analyzing the behavior of users and the post contents on Italian pages dedicated to physiotherapy. There is also an urgent need to evaluate whether virtual communities may improve final patient outcomes.

## Dear Sir,

Since I graduated in June 1997, the tools available for knowledge sharing, interaction, and professional debate among physiotherapists have changed greatly. In those days, to perform the bibliographic research for my thesis I had three possibilities: 1) to consult Index Medicus, a monthly guide to medical articles in thousands of journals where a huge volume of bibliographic citations were manually compiled; 2) through the MEDLINE CD-ROMs, each one containing 6 months of citations (though students could not freely access this valuable resource!); or 3) to physically browse the hundreds of journals in the institute’s scientific library where I was a trainee. In all cases, the probability of accomplishing an exhaustive, comprehensive search was equal to that of looking for a needle in a haystack, and there was no opportunity to interact with scientists. By coincidence, the PubMed system was offered free to the public exactly in June 1997, ushering in the era of private, home-based searching through the Web.

More recently, the advent of social media such as Facebook (FB) has introduced new possibilities for scientific dissemination, potentially expanding the boundaries of knowledge and professional networking beyond the limits of time, physical location, or geography [1]. Unfortunately, it has also opened the door to an undetermined amount of redundant, non-controlled and self-referred information. As of March 31, 2016, FB had more than 1.65 billion monthly active users [2], and in Italy I have recently reviewed at least 15 groups with more than 5000 followers each dedicated exclusively to physiotherapy. This closure to other professions implies a sort of tribal behavior, and may inhibit inter-professional knowledge sharing. But how do Italian physiotherapists use FB to develop their professional networking and knowledge?

Currently there are no data available in Italy, and little is known also on how health care professionals create virtual communities abroad [3]. In particular, there are limited data to describe the type of knowledge exchanged, its accuracy, whether the knowledge supplied answered the question or not, and what users then do with the knowledge shared. However, theoretical frameworks can be proposed by analyzing the posting behaviors and post contents on FB.

The 15 FB groups dedicated to our profession have been followed daily for a 4 week period. The first observation concerns the profile of users: what stands out is the large presence of students and new graduates (sharing in common a positive attitude toward the media) who request specific clinical information but interact with few experienced colleagues. The sharing of knowledge, in theory, should be facilitated by a culture of collectivism, reciprocity, and a respectful noncompetitive environment [1]. Unfortunately, these are traits not commonly observed on Italian FB groups, and credible expert peers - such as those with an active role in universities or scientific societies, or leading authors in scientific journals - demonstrate low posting behaviors. The underlying motivations may vary, but they are probably related at least in part to the content and tone of the posts. Actually, many conversations on clinical posts fail, showing a low level of interaction and of reciprocal influence between posts. Sometimes many answers are given, but they are in contrast with each other or not supported by scientific evidence (or, worse still, not even based on anatomical or physiological knowledge!). It is not uncommon also that the tone becomes intense when contrasting opinions are expressed among users, undermining the ‘professional climate’. Moreover, these negative group behaviors have an undesirable effect on both the willingness to share knowledge and the retention of community members [3, 4].

In some instances, page administrators or “expert” users have obvious conflicts of interest such as teaching of private courses, partnerships with companies, or they are proponents of a specific “school” of rehabilitation theory. Such users can exert a powerful influence on younger fellows through the social media, and such unethical *Facebook-based practice* can easily lead to biased knowledge and a self-referential system akin to ‘guruism’. It is not easy to find a way to counter this problem, but a possible solution would be to invest resources by scientific societies or professional associations. The effective management of FB groups and pages requires massive resources of time, communication skills, and expertise in the field of physical therapy, and it can not be considered as a secondary activity to manage on an occasional basis as often happens.

The proportion of clinical versus nonclinical posts varies greatly across different FB groups, and cannot be generalized. Nonclinical posts are usually related to courses and conferences (unfortunately, these information/advertising posts are often related to private courses of little scientific relevance), medical equipment (such as electromedical devices, tape, etc.), job offers or requests (also relating to work abroad), and discussion on health policy issues, compensation, or abusive practice in rehabilitation. Surprisingly little has been posted on the questionable (to say the least) rules recently used to qualify physiotherapists as professors in the rehabilitation sector of the Italian academia [5, 6]. Less exploited - but potentially of great utility especially for freelancers who have no direct access to university libraries - is the opportunity that FB provides of sharing scientific articles for study purposes or fostering peer collaboration or mentorship.

Web-based forums may facilitate the development of cognitive skills such as students’ critical thinking. Students also frequently ask for help for their dissertations. However, in many cases the questions asked are put too generically and remain unanswered. FB can become a very useful tool in this regard - for example, to run a survey or get expert opinion of colleagues on a particular subject - but its use must be carefully guided by the thesis supervisor.

A virtual community is composed of members who participate at different levels, with a mixture of lurkers, observers, passive, and active contributors [1]. Noncontributing community members are likely to belong because of potential access to important information, but other reasons should be considered and analyzed in future studies. In fact, notwithstanding the positive attitude toward social media in general, a healthy skepticism is always needed regarding the truth of the information.

Internet has revolutionized our way of communicating, and there are currently opposing views regarding the benefits and pitfalls of professional use of social media. The creation and development of multidisciplinary networks should be encouraged as they can be effective in facilitating the transfer of experience and research knowledge across professional boundaries. However, further research is strongly needed to evaluate whether virtual communities may improve final patient outcomes.

## Abbreviations

FB: Facebook
